# National antibiotic consumption is strongly related to the prevalence of antibiotic resistance across bacterial clades

**DOI:** 10.1016/j.isci.2024.111712

**Published:** 2025-01-06

**Authors:** Stilianos Louca

**Affiliations:** 1 Department of Biology, University of Oregon, Eugene, OR 97403, USA; 2 Institute of Ecology and Evolution, University of Oregon, Eugene, OR 97403, USA

**Keywords:** natural sciences, biological sciences, microbiology, clinical microbiology

## Abstract

The impact of societal antibiotic consumption on the prevalence of antibiotic resistance across microbial taxa in natural environments has not yet been assessed at global scales. Here, I examine the prevalence of 155 antibiotic resistance genes (ARGs) in 300,209 bacterial genomes, from non-clinical non-human-associated terrestrial environments at over 9,600 locations in 44 countries. I then compare ARG prevalences to nationwide antibiotic consumption rates, distinguishing between different ARG types. I find that depending on country and ARG type, ARG prevalences can be extremely high; for example, the probability that a given quinolone resistance gene is present in a given strain in Thailand was estimated at 42%. Further, I find strong positive correlations between nationwide antibiotic consumption rates and mean ARG prevalences for nearly all ARG types. Thus, national antibiotic consumption leaves a signal on the prevalence of ARGs across the bacterial tree, even in non-clinical environments.

## Highlights

• Prevalence of antibiotic resistance genes (ARGs) assessed in >300,000 bacterial genomes
• Depending on country and ARG type, ARG prevalence can be extremely high
• ARG prevalences correlate strongly with national antibiotic consumption rates

## Graphical abstract

**Figure:**
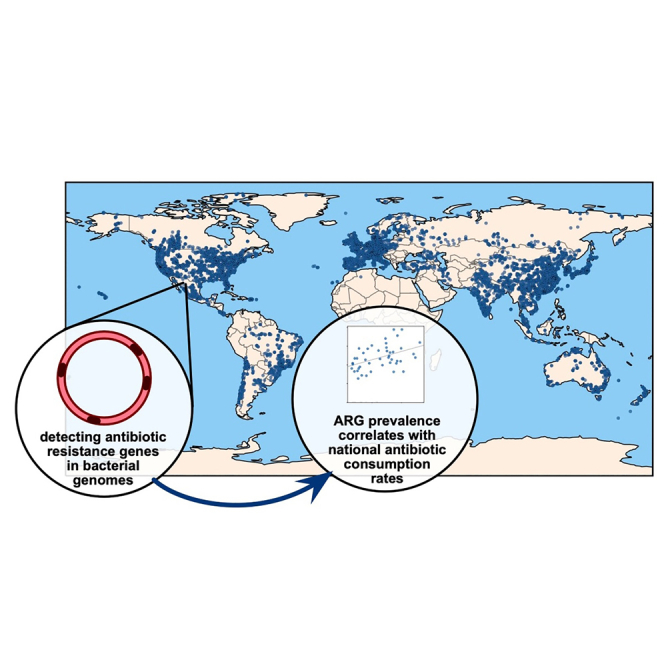

## Introduction

The rapid spread of antibiotic resistance among microorganisms, believed to be largely driven by an increased use of antibiotics, is a topic of great concern.^1^^,^^2^ Numerous outbreaks of strains resistant to common antibiotics have been documented throughout the world,^3^^,^^4^^,^^5^^,^^6^ and a causal relationship between antibiotic use and acquired antibiotic resistance in clinical settings and in patients is well established.^7^^,^^8^^,^^9^^,^^10^^,^^11^ Metagenomic sequencing studies have also revealed a wide distribution of antibiotic resistance-conferring genes (ARGs) in non-clinical local environments such as lakes, wastewater, aquaculture, and soil,^12^^,^^13^^,^^14^^,^^15^^,^^16^^,^^17^ suggesting that unintended leakage or even intentional disposal of antibiotics into the environment may drive the proliferation of ARGs. However, ARGs in the environment are not strictly a human-induced phenomenon, since selective pressure for ARGs likely also existed in ancient microorganisms^12^^,^^18^^,^^19^^,^^20^; for example, antibiotic resistance can be an adaptive trait involved in competition between microorganisms.^21^^,^^22^ Further, antibiotic resistance can also be selected by other pollutants or naturally occurring chemicals, notably heavy metals, through various mechanisms including co-resistance or cross-resistance.^20^^,^^23^^,^^24^ For example, antibiotic resistance in bacteria, sampled along a sediment archive as far back as the 19th century, correlated positively with zinc concentrations over time.^20^ Hence, it is important to understand the extent to which antibiotic resistance in the non-clinical environment and across microbial clades is driven by human antibiotic use. Such insights are particularly important in light of ongoing efforts to regulate the use of antibiotics.^25^ Previous comparisons between antibiotic consumption and microbial resistance to antibiotics over large geographic scales have focused on a small number of well-studied cultured organisms.^26^^,^^27^^,^^28^^,^^29^^,^^30^ To date, an evaluation of ARG prevalence in microbial taxa from non-clinical environments, covering a broad taxonomic spectrum, and its potential relationship with national antibiotic consumption rates is lacking. Evaluating this relationship at a national level is particularly important, since most laws regulating antibiotic use are national.

Here, I examine the prevalence of 155 ARGs in 300,209 publicly available bacterial medium- to high-quality genomes (>50% completion, <5% contamination) from non-clinical non-human microbiome terrestrial environments, recovered using both culture-based and culture-independent methods. The genomes originate from over 27,700 studies in 44 countries, cover over 9,600 geographic locations worldwide including soils, lakes, rivers, aquifers, bioreactors, farms, livestock, wildlife, plants, and hot springs, and represent approximately 240 bacterial phyla (overview of taxa in Figure S1, accession numbers in Data S1). I distinguish between genes involved in various types of antibiotic resistance, including beta-lactamases as well as resistance to aminoglycosides, fosfomycins, macrolide-lincosamide-streptogramin (MLS) antibiotics, phenicols, quinolones, rifamycins, sulfonamides, tetracyclines, trimethoprim, and vancomycin. I use a maximum likelihood approach under a Poisson binomial distribution model to estimate gene prevalences across organisms, while accounting for the incompleteness of some genomes. I then compare the estimated nationwide ARG prevalences in each country to the national antibiotic consumption rate during the years 2000–2016, measured in defined daily dose equivalents per year. As described further, I find strong and significant statistical associations between the national antibiotic consumption rate and ARG prevalences across countries.

## Results and discussion

### ARG prevalences across countries and cells

The prevalence of a given ARG is henceforth defined as the fraction of all examined strains that exhibit the gene, in other words, the probability that any given cell exhibits that gene. This quantity was estimated statistically based on the presence/absence of the gene in the recovered genomes and accounting for the completeness of those genomes. ARG prevalences were either estimated separately for genomes from each country or estimated using all 300,209 genomes from all considered countries. To limit estimation error, only countries with at least 500 genomes were considered (44 countries; Figure S2). For simplicity, and to reduce estimation noise, I focus on mean ARG prevalences, either averaged over all 155 ARGs or averaged over all ARGs of a particular type (e.g., average ARG of all fosfomycin resistance genes).

**Figure 1:**
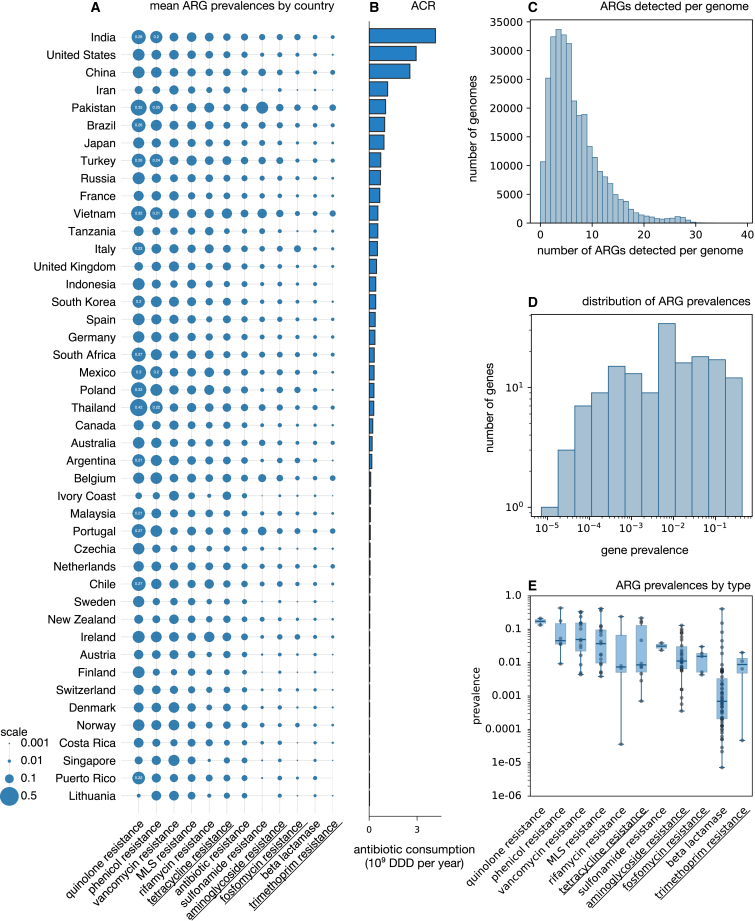
ARG prevalences (A) Mean estimated prevalences of antibiotic resistance genes (ARG) in analyzed bacterial strains, for each country (rows) and each gene type (columns). Circle sizes are proportional to mean ARG prevalence, i.e., the fraction of cells exhibiting any given ARG averaged over all ARGs of a specific type. Values above 0.2 are written inside the circles. ARG types conferring resistance to antibiotics classified as “reserve” by the WHO AWaRe system^31^ are underlined. (B) National antibiotic consumption rate (ACR, daily dose equivalents per year) per country. (C) Histogram of the number of ARGs detected in individual genomes, not adjusted for genome completeness (each genome is one data point). (D) Histogram of ARG prevalences (each considered ARG is one data point). (E) Estimated prevalences of individual ARGs by type (one point per gene, one box per gene type). Whiskers show full value ranges, boxes show interquartile ranges, and horizontal lines show medians. ARG types are sorted by mean prevalence. For analogous plots considering only genomes from human pathogens, or only from non-human pathogens, see Figures S3 and S4, respectively. Also see Figure S5.

Estimated ARG prevalences varied strongly between individual genes, with some genes ranging below 0.01% and some reaching over 30% (Figures 1D and 1E). The average prevalence of all ARGs, that is, the probability that any given ARG gene is found in any given organism, was 6.4%. Given that 155 genes were considered, this implies that any randomly chosen cell is expected to contain on average 9.9 ARGs (155×6.4%). In fact, a substantial portion of examined genomes contained over 10 ARGs (Figure 1C). This indicates that multi-faceted antibiotic resistance is common outside of the clinical environment. ARG types with the highest gene prevalences were associated with quinolone, phenicol, vancomycin, and MLS resistance, while the least prevalent types were beta-lactamases and genes involved in trimethoprim resistance (Figures 1A–1E). ARGs conferring resistance to “reserve” antibiotics sensu World Health Organization (WHO),^31^ that is, antibiotics reserved as last resorts and restricted to cases with no other treatment options, generally exhibited lower prevalences, with trimethoprim and fosfomycin resistance genes being the least or 3rd least prevalent. ARG prevalences also varied substantially between countries (Figure 1A). Notably, ARG prevalence was above 30% for quinolone resistance genes in multiple countries, including Thailand (42%), Pakistan (35%), Poland (33%), and Vietnam (32%). Thus, a randomly chosen gene involved in quinolone resistance would have a 42% probability of being in any randomly chosen strain in Thailand, or a 35% probability for Pakistan, and so on.

Note that different ARG types can comprise very different numbers of genes; for example, across all genomes, I detected 60 distinct beta-lactamase genes and only 2 distinct quinolone genes (Table S1). Thus, differences in average prevalences between ARG types must be interpreted with caution. Indeed, when considering the sum of ARG prevalences within each ARG type, that is, the expected number of ARGs per cell and per ARG type, vancomycin and MLS resistance genes were generally the most abundant (Figure S5). The highest expectation was estimated for vancomycin resistance (2.4 genes per cell in Singapore) and beta-lactamases (2.4 genes per cell in Pakistan).

To further examine ARG prevalence in human pathogens, a topic of particular concern, I repeated the aforementioned analyses separately for genomes from species known to be obligate or opportunistic human pathogens, henceforth simply referred to as “pathogens” (48,509 genomes, Figure S3). Note that since this constitutes a small subset of the original genomic dataset, a sufficient number of genomes for analysis were only available for a subset of 20 countries. Across this subset of countries, the average ARG prevalence was 3.9%. I thus found no evidence that ARG prevalence is higher in pathogens sampled from non-clinical non-human microbiome environments, when compared to bacteria overall. The two ARG types with highest gene prevalences were quinolone resistance and phenicol resistance, with the highest prevalence estimated for quinolone resistance in Thailand (43%), similarly to the full dataset. When considering the expected number of ARGs per cell and per ARG type (Figure S5), the highest value was estimated for beta-lactamases in Russia (2.8 genes per cell) and aminoglycoside resistance in China (2.8 genes per cell). Given that some aminoglycosides are classified as “reserve” or “watch”’ antibiotics by WHO,^31^ these high values appear particularly worrisome.

### National antibiotic consumption rate versus ARG prevalence

**Figure 2:**
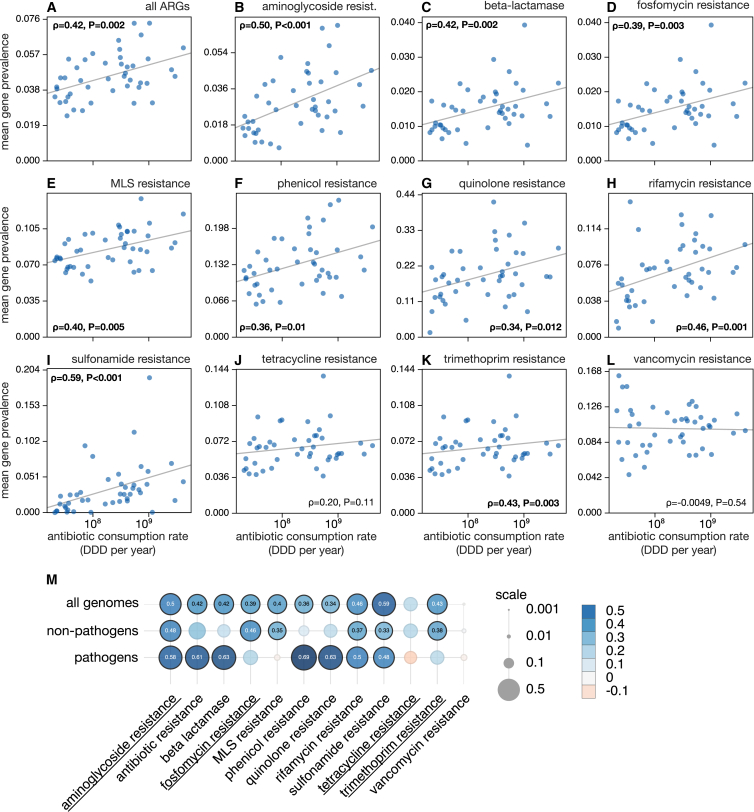
ARG prevalences versus antibiotic consumption rates (A) Mean estimated ARG prevalence (fraction of cells exhibiting a given gene, averaged over all genes, vertical axis) compared to national antibiotic consumption rate (daily dose equivalents per year, horizontal axis) across 44 countries (one point per country). (B–L) Similar to (A), but focusing on specific categories of ARGs. In all figures, the Spearman rank correlation (ρ) and its associated one-sided statistical significance (*p*) are shown; bold letters highlight statistically significant cases. Log-linear least-squares regression lines are shown for reference. For analogous plots considering only genomes from human pathogens, or only from non-human pathogens, see Figures S6 and S7, respectively. (M) Spearman correlations between mean ARG prevalences and national antibiotic consumption rates, considering either genomes identified as human pathogens, genomes not identified as human pathogens, or all genomes. Circle sizes are proportional to the magnitude of correlation coefficients. Statistically significant correlations are highlighted using a black ring around the circle and an inscription of the correlation coefficient. ARG types conferring resistance to antibiotics classified as “reserve” by the WHO AWaRe system^31^ are underlined.

To examine whether antibiotic consumption rates have an effect on ARG prevalence at a national scale, I compared mean ARG prevalences to each country’s antibiotic consumption rate, estimated for the period 2000–2016 by averaging data from various years in that interval (Figure 2).^25^^,^^32^ I found a strong and significant positive Spearman correlation between mean ARG prevalence and antibiotic consumption rate across countries for (ρ=0.42, one-sided *p* = 0.002). Similarly, strong and significant positive correlations were also found for 9 out of 11 individual ARG types: beta-lactamases and resistance to aminoglycosides, fosfomycins, MLS, phenicols, quinolones, rifamycins, sulfonamides, and trimethoprim. This suggests that societal consumption of antibiotics has a clearly detectable and substantial influence on the abundance of ARGs across microbial taxa within national boundaries, in non-clinical non-human microbiome environments. Correlations were particularly strong for genes involved in sulfonamide resistance (ρ=0.59, *p* < 0.001) and aminoglycoside resistance (ρ=0.50, *p* < 0.001). Sulfonamides are among the oldest broadly used antibacterials, whose popularity dates back prior to the introduction of penicillin.^33^ Aminoglycosides are broad-spectrum antibiotics with a primarily clinical use, dating back to the 1940s.^34^ The present results suggest that relative to other (natural) factors, human consumption of antibiotics has a particularly strong effect on the taxonomic distribution of sulfonamide and aminoglycoside resistance genes.

On the other end, mean prevalences of ARGs involved in tetracycline and vancomycin resistance did not exhibit a significant correlation to national antibiotic consumption rates. The lack of a significant correlation for these two ARG types may reflect a genuinely weak sensitivity of bacterial evolutionary/ecological dynamics to these drugs. Alternatively, it is possible that the national consumption specifically of tetracyclines and vancomycins is poorly correlated with overall national antibiotic consumption, which was the only metric of antibiotic use easily available for this study. In other words, while the prevalence of tetracycline and vancomycin resistance genes may indeed be affected by the presence of these drugs, the usage rates of the latter may not be well reflected by overall antibiotic consumption rates. Indeed, it is likely that the relative contributions of various types of antibiotics to national antibiotic consumption differ between countries, for example, depending on local regulations and agricultural activities.^32^^,^^35^

To further examine the potential effects of antibiotic consumption on pathogens, I repeated the aforementioned analyses focusing on genomes identified as human pathogens (48,509 genomes across 20 countries). In general, correlations between ARG prevalences and national antibiotic consumption rates were similarly strong or stronger than for the full dataset. (Figures 2M and S6), with the exception of fosfomycin and trimethoprim resistance for which no significant correlations were found. Notably, correlation coefficients were as high as 0.69 for phenicol resistance (*p* = 0.003), 0.63 for beta-lactamases (*p* = 0.001), and 0.63 for quinolone resistance (*p* = 0.001). When analyzing only non-pathogen genomes (243,026 genomes across 36 countries), significant correlations were observed for 6 out of 11 ARG types. ARG types for which correlations were significant in the full dataset but non-significant in non-pathogens (beta-lactamases, phenicol resistance, and quinolone resistance) were the ARG types with strongest correlations in pathogens. These results suggest that some types of antibiotics exert a detectably stronger selective pressure on pathogens, when compared to other antibiotics or when compared to non-pathogens. It should be pointed out, however, that this conclusion does not apply to some ARG types, such as fosfomycin and MLS resistance, where pathogens displayed weak and non-significant correlations.

The aforementioned results contrast previous much smaller studies that detected no significant association, or only a weak association, between national antibiotic consumption rates and ARG prevalences.^26^^,^^27^^,^^28^^,^^29^ This difference may be explained by the fact that each of those studies focused only on a small number of clinically relevant taxa, notably *Escherichia coli*, *Klebsiella* spp., and *Staphylococcus aureus*. Hence, while national antibiotic consumption rate strongly correlates with ARG prevalence when assessed over the full bacterial domain, it need not necessarily correlate with ARG prevalence in every single bacterial species.

### Limitations of the study

A few caveats are worth noting. First, the present analysis does not clarify whether elevated ARG prevalence in some countries is primarily driven by horizontal transfers and fixation of these genes in new taxa, or primarily through the invasion by foreign strains that already exhibit these genes. Distinguishing between these two scenarios is difficult and would require analysis of the recent phylogenetic history of ARG alleles or, hypothetically, an experimental modification of microbial dispersal across countries. I also emphasize that antibiotic resistance can additionally be enhanced through mutations in general-purpose genes, for example, mutations affecting the cell wall’s permeability to small molecules.^36^ Such forms of antibiotic resistance are not covered by the present analysis, which focuses on genes primarily associated with antibiotic resistance. Further, ARGs are often transported on plasmids^37^ and are not necessarily immediately incorporated into chromosomes. Since plasmids are sometimes missing from published genomes, especially those assembled from metagenomes,^38^ ARG prevalences reported here may in some cases underestimate the true extent of antibiotic resistance. Lastly, the unavoidable “blurry” temporal aspect of the patterns examined and the data used should be kept in mind. Specifically, national antibiotic consumption in any given year could affect ARG prevalences over multiple subsequent years, and reciprocally, ARG prevalence at any given time could be cumulatively affected by antibiotic consumption over multiple preceding years.^39^ Similarly, the examined national antibiotic consumption rates, as well as the genomes used to estimate ARG prevalences, cover a broad time interval, rather than a single snapshot. Thus, both the ARG prevalences and the compared antibiotic consumption rates should be seen as approximate averages over time. Given that such an averaging would generally tend to hide causal relationships and deflate correlations, the strong correlations reported here are particularly remarkable.

### Conclusions

This study analyzed hundreds of thousands of bacterial genomes worldwide, from non-clinical non-human microbiome environments, to examine the proliferation of antibiotic resistance genes across the bacterial tree of life and to assess the role of national antibiotic consumption rate in as a potential driver of this proliferation. It was found that the prevalence of some ARGs was extraordinarily high in some countries (e.g., >40% in Thailand) and that a substantial fraction of strains exhibit over 10 different ARGs. It was further shown that national antibiotic consumption rate exhibits a strong positive correlation with ARG prevalence across countries. The latter observation held for the majority of ARG types examined, in pathogens as well as in non-pathogens. These correlations are particularly remarkable if one considers the inevitable differences in regulations, antibiotic usage streams, and environmental conditions between countries, all of which would tend to conceal the underlying causal relationships. The present study substantially expands analogous findings from previous studies that focused on specific bacterial species^26^^,^^27^^,^^28^^,^^29^^,^^30^ and studies reporting a relationship between local ARG prevalence and antibiotics in specific geographic regions.^40^^,^^41^ The findings presented here suggest that antibiotic consumption at the national level substantially impacts ARG prevalence in the environment, and they underline the urgency to develop national policies that curb the overuse of antibiotics.^42^ In particular, it becomes clear that a reduction of antibiotic consumption at the national level not only serves a global “public good”^43^ but would also decrease ARG prevalence in the specific countries or regions enacting such reductions.

## Resource availability

### Lead contact

Reasonable requests for further information and resources should be directed to and will be fulfilled by the lead contact, Stilianos Louca (louca.research@gmail.com).

### Materials availability

This study did not generate new samples or unique reagents.

### Data and code availability

• All data have been published previously and are available at locations described in the Methods. Accession numbers for GenBank genomes are given in Data S1. Country metadata, including antibiotic consumption rates, are listed in Data S3.
• No novel code was generated in this study. All software used in this paper have been described in the Methods and are freely available online.
• Any additional information required to reanalyze the data reported in this paper is available from the lead contact upon request.

## Acknowledgments

S.L. was supported by a startup grant by the 10.13039/100011348University of Oregon.

## Author contributions

S.L. performed all analyses and wrote the manuscript.

## Declaration of interests

The author declares no competing interests.

## STAR★Methods

### Key resources table

**Table:** 

REAGENT or RESOURCE	SOURCE	IDENTIFIER
**Software and algorithms**		

R	R project	https://www.r-project.org
python	Python project	https://www.python.org

**Other**		

Bacterial genomes in fasta format	NCBI GenBank	Data S1
Bacterial genomes in fasta format	Genomes from Earth’s Microbiomes (GEM)	Data S1

### Method details

#### Genomes

In the following, the term “genome” refers to complete or incomplete genome sequences obtained from culture-dependent as well as culture-independent methods, including metagenome-assembled genomes.^44^ Genomes were obtained from two different sources, GenBank^45^ and the Genomes from Earth’s Microbiomes (GEM) catalog,^46^ as follows. Complete or draft genome sequences were downloaded from GenBank^45^ on April 26, 2024, based on the following criteria: Only genomes with a contig-N50 above 5000, and with the “excluded_from_refseq” entry either being empty or only containing the terms “*na*”, “*not used as type*”, “*untrustworthy as type*”, “*derived from metagenome*”, “*missing rRNA genes*”, “*missing tRNA genes*”, “*derived from environmental source*”, “*derived from single cell*”, “*unverified source organism*”, “*partial*” and “*genus undefined*” were downloaded. The original sample coordinates were extracted from the corresponding BioSample’s “latitude”, “longitude” and/or “lat_lon” fields or (for a small number of isolate genomes) from the literature. GEM genomes and associated metadata were downloaded from the official repository (https://genome.jgi.doe.gov/portal/GEMs/GEMs.home.html) on April 17, 2021, and only genomes with available coordinates were kept. The country of each genome was either determined based on its coordinates if available, using the python package reverse_geocoder v1.5.1, or extracted from the geo_loc field in the NCBI sample metadata. Genomes for which the country of origin could not be determined were omitted. The taxonomic identities of GenBank genomes were taken from GenBank based on each genome’s taxid, while the taxonomic identities of GEM genomes were taken from the provided metadata.

Genomes were associated with various environments as follows. GenBank genomes were classified as human-microbiome-associated if their BioSample’s “host” entry was “*Homo sapiens*” or their “isolation_source” entry contained the word “*Homo sapiens*” or “*human*”. GEM genomes were classified as human-microbiome-associated if their “habitat” metadata started with “*human host*”, or if their “ecosystem_type” metadata was “*human*”. Genomes were identified as human pathogens using the FAPROTAX database v1.2.4^47^ based on their taxonomic identity, including for example *Cutibacterium acnes*, *Eggerthella lenta*, *Mycobacterium tuberculosis*, *Bacillus anthracis*, *Staphylococcus aureus*, *Enterococcus faecalis*, *Listeria monocytogenes* and several others. Genomes were identified as marine either based on their coordinates (if available) using the python package global_land_mask v0.0.3, or based on the presence of one of the following keywords in their sample metadata: “*marine*”, “*ocean*”, “*seafloor*”, “*seawater*”. Genomes were identified as terrestrial if they were not marine, and only terrestrial genomes were kept for further analysis. Terrestrial GenBank genomes were classified as originating in a clinical setting if their BioSample’s “geo_loc”, “metagenome-source”, “organism_name”, “isolation_source”, “isolation_site” or “env_local_scale” entry contained “*hospital*” or “*clinic*”. Terrestrial GEM genomes were classified as originating in a clinical setting if their “habitat” or “ecosystem_type” metadata contained “*hospital*” or “*clinic*”. In all subsequent analysis, I focused on terrestrial non-clinical non-human-microbiome-associated genomes, occasionally distinguishing between human pathogens and non human pathogens.

Proteins were predicted for each genome using prodigal v2.6.3.^48^ The quality of each genome was assessed based on the presence of multiple single-copy marker genes using checkM v1.1.3,^49^ with option “reduced_tree”. Only genomes with an estimated completeness ≥50% and a contamination level ≤5% were kept, and only countries with at least 500 quality-filtered genomes and available information on total annual antibiotic consumption rates (see below) were considered for subsequent analyses. Hence, we obtained a dataset of 300,209 medium- to high-quality georeferenced terrestrial non-clinical non-human-microbiome-associated genomes from 44 countries, with an average completeness of 92.7% and an average contamination level of 0.98%. When considering only genomes from human pathogens, 20 countries were kept based on the above criteria, represented by 48,509 genomes. When considering only genomes not from human pathogens, 36 countries were kept based on the above criteria, represented by 243,026 genomes. A map of geographic locations of genomes (wherever available) and overviews of taxonomic coverages (genomes per phylum or class) are shown in Figure S1. An overview of the number of genomes from each country is shown in Figure S2C. Genome accession numbers, taxonomic classifications, coordinates (where available), country of origin, completeness, contamination levels, pathogen identifications and other metadata are provided in Data S1. Readers can retrieve any of the GenBank genomes analyzed based on its accession number, under a URL of the form https://www.ncbi.nlm.nih.gov/datasets/genome/GCA_030518215.1, after replacing GCA_030518215.1 with the proper accession number. Readers can also retrieve information on a genome’s original project based on its bioproject number (provided in Data S1), under a URL of the form https://www.ncbi.nlm.nih.gov/bioproject/PRJNA988455, after replacing PRJNA988455 with the proper bioproject number.

#### National antibiotic consumption rates

National antibiotic consumptions per day and per person (in daily dose equivalents, DDD) for the period 2000–2016 were obtained from the literature or estimated, as follow. For countries covered by the WHO Report of Surveillance of Antibiotic Consumption,^25^ the DDD per day per person for the years 2015 or 2016 was taken from that report (table 4.2 therein). Additional antibiotic consumption data (DDD per day per person) for the years 2000, 2015 and/or 2005 were taken from Klein et al.*,*^32^ supplementary table 3, by dividing the values in the column “*Access antibiotics volume in DID*” by the values in the column “*Access antibiotics in total consumption*” and further dividing by 1000. National antibiotic consumption rates (ACR, in DDD per year) were calculated by multiplying the daily per person consumption rates by 365 and by the country’s population size in the corresponding year. The population size of each country in each considered year was obtained from the World Bank Development Indicators database (https://datatopics.worldbank.org/world-development-indicators, accessed October 15, 2024). For each country, available national antibiotic consumption rates were averaged to obtain a single numeric value representative of the period 2000–2016. A temporal average was preferred over any single time point, to reduce sensitivity to events in any single year and to account for the fact that ARG prevalences too were estimated using genomes collected over multiple years. For a comparison of antibiotic consumption rates between the 3 most represented years, see Figure Figure S2. Overall, a strong and significant positive correlation was observed in antibiotic consumption rates between years (Spearman rank correlation ≥ 0.95 in all cases, *p* < 0.05). Information on each considered country, including population sizes and antibiotic consumption rates, is provided in Data S3.

### Quantification and statistical analysis

#### Prevalence of antibiotic resistance genes

Throughout this article, the “prevalence” of a given gene is defined as the fraction of cells exhibiting the gene, either in the whole genomic dataset or restricted to genomes from a specific country and optionally further restricted to pathogens or non-pathogens. To estimate the prevalence of antibiotic resistance genes (ARGs), I proceeded as follows. Genes were initially chosen according to the catalog of antibiotic resistance genes in the KEGG BRITE database,^50^ classification BR1600, however only genes found in at least one genome were ultimately considered. A table listing the 155 considered genes, including names, KOs and descriptions, is given in Data S2, and an overview is given in Table S1. Protein coding genes were predicted using prodigal as described above, and functionally annotated against the KOfam Hidden Markov Model (HMM) database v95.0, released 2020-09-06,^51^ using hmmsearch v3.3.^52^ Only hits with a score above an HMM’s provided noise cutoff and an E-value below $10^{-10}$ were accepted. For each genome, a gene was considered to be present if the corresponding HMM was represented by at least one accepted hit. Note that in the case of incomplete genomes, a gene may be absent from the genome sequence even if it was present in the actual cells that the genome represents. Thus, simply counting the fraction of genomes in which a gene was found would underestimate the actual prevalence of the gene. In order to estimate a gene’s prevalence while accounting for genome completeness, a maximum-likelihood approach based on a Poisson binomial distribution model was used. In this model, a given gene has probability $C_{i}$ of being detected in the i-th genome, when conditioned on the gene being present in the original cells, where $C_{i}$ is the genome’s completeness (estimated with checkM as explained earlier). False positive detections were assumed to be negligible. Let $M_{0}$ denote the subset of considered genomes in which the gene was not detected, and $M_{1}$ the subset of considered genomes in which the gene was detected. Let α be the gene’s prevalence, i.e., the unknown parameter to be estimated. Then according to the model, for any given α the likelihood of the data is given by:

(Equation 1) $$L=\prod_{i\in M_{0}}\left[\left(1-\alpha \right)+\alpha \left(1-C_{i}\right)\right]\times \prod_{i\in M_{1}}\alpha C_{i}\text{.}$$

By taking the first derivative of the log likelihood with respect to α and demanding that it be zero (i.e., dln(L)/dα=0), we obtain the following condition for the maximum-likelihood estimate $\hat{\alpha }$:

(Equation 2) $$0=\frac{\left|M_{1}\right|}{\hat{\alpha }}-\sum_{i\in M_{0}}\frac{C_{i}}{1-\hat{\alpha }C_{i}}\text{,}$$

where $\left|M_{1}\right|$ denotes the size of $M_{1}$, i.e. the number of genomes where the gene was detected. Equation 2 was solved numerically using the bisection method implemented in the python function scipy.optimize.root_scalar.^53^ The above procedure was applied separately for each gene, and separately for each subset of considered genomes (e.g., genomes from India, genomes from the USA, and so on). Estimated AGR prevalences in each country are given in Data S3. The numbers of ARGs found in each genome and for each ARG type are provided in Data S1.

## Supplemental information

Document S1. Figures S1–S7 and Table S1

Data S1. Genome metadata, including accession numbers, related to STAR Methods

Data S2. Summaries per gene, including estimated prevalences, related to Figure

Data S3. Country metadata, including antibiotic consumption rates, related to STAR Methods

## References

[1] Neu H.C. (1992). The crisis in antibiotic resistance. Science.

[2] Bush K., Courvalin P., Dantas G., Davies J., Eisenstein B., Huovinen P., Jacoby G.A., Kishony R., Kreiswirth B.N., Kutter E. (2011). Tackling antibiotic resistance. Nat. Rev. Microbiol..

[3] Finland M. (1979). Emergence of antibiotic resistance in hospitals, 1935–1975. Rev. Infect. Dis..

[4] Lipsitch M., Bergstrom C.T., Levin B.R. (2000). The epidemiology of antibiotic resistance in hospitals: paradoxes and prescriptions. Proc. Natl. Acad. Sci. USA.

[5] Malande O.O., Du Plessis A., Rip D., Bamford C., Eley B. (2016). Invasive carbapenem-resistant enterobacteriaceae infection at a paediatric hospital: A case series. S. Afr. Med. J..

[6] Tshisevhe V.S., Lekalakala M.R., Tshuma N., Janse van Rensburg S., Mbelle N. (2016). Outbreak of carbapenem-resistant *Providencia rettgeri* in a tertiary hospital. S. Afr. Med. J..

[7] Miller G.H., Sabatelli F.J., Hare R.S., Glupczynski Y., Mackey P., Shlaes D., Shimizu K., Shaw K.J. (1997). The most frequent aminoglycoside resistance mechanisms–Changes with time and geographic area: A reflection of aminoglycoside usage patterns?. Clin. Infect. Dis..

[8] Gaynes R., Monnet D. (2007).

[9] Davies J., Davies D. (2010). Origins and evolution of antibiotic resistance. Microbiol. Mol. Biol. Rev..

[10] Chatterjee A., Modarai M., Naylor N.R., Boyd S.E., Atun R., Barlow J., Holmes A.H., Johnson A., Robotham J.V. (2018). Quantifying drivers of antibiotic resistance in humans: a systematic review. Lancet Infect. Dis..

[11] Perry M.R., Lepper H.C., McNally L., Wee B.A., Munk P., Warr A., Moore B., Kalima P., Philip C., de Roda Husman A.M. (2021). Secrets of the hospital underbelly: patterns of abundance of antimicrobial resistance genes in hospital wastewater vary by specific antimicrobial and bacterial family. medRxiv.

[12] Allen H.K., Donato J., Wang H.H., Cloud-Hansen K.A., Davies J., Handelsman J. (2010). Call of the wild: antibiotic resistance genes in natural environments. Nat. Rev. Microbiol..

[13] Chen Q., An X., Li H., Su J., Ma Y., Zhu Y.G. (2016). Long-term field application of sewage sludge increases the abundance of antibiotic resistance genes in soil. Environ. Int..

[14] Manaia C.M., Rocha J., Scaccia N., Marano R., Radu E., Biancullo F., Cerqueira F., Fortunato G., Iakovides I.C., Zammit I. (2018). Antibiotic resistance in wastewater treatment plants: tackling the black box. Environ. Int..

[15] Huang Z., Zhao W., Xu T., Zheng B., Yin D. (2019). Occurrence and distribution of antibiotic resistance genes in the water and sediments of Qingcaosha Reservoir, Shanghai, China. Environ. Sci. Eur..

[16] Shen X., Jin G., Zhao Y., Shao X. (2020). Prevalence and distribution analysis of antibiotic resistance genes in a large-scale aquaculture environment. Sci. Total Environ..

[17] Martiny H.M., Munk P., Brinch C., Aarestrup F.M., Petersen T.N. (2022). A curated data resource of 214K metagenomes for characterization of the global antimicrobial resistome. PLoS Biol..

[18] D’Costa V.M., King C.E., Kalan L., Morar M., Sung W.W.L., Schwarz C., Froese D., Zazula G., Calmels F., Debruyne R. (2011). Antibiotic resistance is ancient. Nature.

[19] Perry J., Waglechner N., Wright G. (2016). The prehistory of antibiotic resistance. Cold Spring Harb. Perspect. Med..

[20] Dickinson A.W., Power A., Hansen M.G., Brandt K.K., Piliposian G., Appleby P., O'Neill P.A., Jones R.T., Sierocinski P., Koskella B., Vos M. (2019). Heavy metal pollution and co-selection for antibiotic resistance: A microbial palaeontology approach. Environ. Int..

[21] Baba T., Schneewind O. (1998). Instruments of microbial warfare: Bacteriocin synthesis, toxicity and immunity. Trends Microbiol..

[22] Czárán T.L., Hoekstra R.F., Pagie L. (2002). Chemical warfare between microbes promotes biodiversity. Proc. Natl. Acad. Sci. USA.

[23] Di Cesare A., Eckert E., Corno G. (2016). Co-selection of antibiotic and heavy metal resistance in freshwater bacteria. J. Limnol..

[24] Vats P., Kaur U.J., Rishi P. (2022). Heavy metal-induced selection and proliferation of antibiotic resistance: A review. J. Appl. Microbiol..

[25] WHO (2018).

[26] Collignon P., Athukorala P.c., Senanayake S., Khan F. (2015). Antimicrobial resistance: The major contribution of poor governance and corruption to this growing problem. PLoS One.

[27] Collignon P., Beggs J.J., Walsh T.R., Gandra S., Laxminarayan R. (2018). Anthropological and socioeconomic factors contributing to global antimicrobial resistance: a univariate and multivariable analysis. Lancet Planet. Health.

[28] Collignon P., Beggs J.J. (2019). Socioeconomic enablers for contagion: Factors impelling the antimicrobial resistance epidemic. Antibiotics.

[29] McGough S.F., MacFadden D.R., Hattab M.W., Mølbak K., Santillana M. (2020). Rates of increase of antibiotic resistance and ambient temperature in Europe: a cross-national analysis of 28 countries between 2000 and 2016. Euro Surveill..

[30] Ajulo S., Awosile B. (2024). Global antimicrobial resistance and use surveillance system (GLASS 2022): Investigating the relationship between antimicrobial resistance and antimicrobial consumption data across the participating countries. PLoS One.

[31] (2022).

[32] Klein E.Y., Milkowska-Shibata M., Tseng K.K., Sharland M., Gandra S., Pulcini C., Laxminarayan R. (2021). Assessment of WHO antibiotic consumption and access targets in 76 countries, 2000–15: an analysis of pharmaceutical sales data. Lancet Infect. Dis..

[33] Christensen S.B. (2021). Drugs that changed society: History and current status of the early antibiotics: Salvarsan, sulfonamides, and β-lactams. Molecules.

[34] Krause K.M., Serio A.W., Kane T.R., Connolly L.E. (2016). Aminoglycosides: An overview. Cold Spring Harbor Perspect. Méd..

[35] Hsia Y., Lee B.R., Versporten A., Yang Y., Bielicki J., Jackson C., Newland J., Goossens H., Magrini N., Sharland M., GARPEC and Global-PPS networks (2019). Use of the WHO Access, Watch, and Reserve classification to define patterns of hospital antibiotic use (AWaRe): an analysis of paediatric survey data from 56 countries. Lancet Global Health.

[36] Garneau-Tsodikova S., Labby K.J. (2016). Mechanisms of resistance to aminoglycoside antibiotics: overview and perspectives. Medchemcomm.

[37] Carattoli A. (2013). Plasmids and the spread of resistance. Int. J. Med. Microbiol..

[38] Maguire F., Jia B., Gray K.L., Lau W.Y.V., Beiko R.G., Brinkman F.S.L. (2020). Metagenome-assembled genome binning methods with short reads disproportionately fail for plasmids and genomic islands. Microb. Genom..

[39] Martinez J.L. (2009). Environmental pollution by antibiotics and by antibiotic resistance determinants. Environ. Pollut..

[40] Baquero F., Martínez J.L., Cantón R. (2008). Antibiotics and antibiotic resistance in water environments. Curr. Opin. Biotechnol..

[41] Wang Z., Han M., Li E., Liu X., Wei H., Yang C., Lu S., Ning K. (2020). Distribution of antibiotic resistance genes in an agriculturally disturbed lake in china: Their links with microbial communities, antibiotics, and water quality. J. Hazard Mater..

[42] Lim J.M., Singh S.R., Duong M.C., Legido-Quigley H., Hsu L.Y., Tam C.C. (2020). Impact of national interventions to promote responsible antibiotic use: a systematic review. J. Antimicrob. Chemother..

[43] Hoffman S.J., Røttingen J.A., Frenk J. (2015). International law has a role to play in addressing antibiotic resistance. J. Law Med. Ethics.

[44] Sharon I., Banfield J.F. (2013). Genomes from metagenomics. Science.

[45] Clark K., Karsch-Mizrachi I., Lipman D.J., Ostell J., Sayers E.W. (2016). Genbank. Nucleic Acids Res..

[46] Nayfach S., Roux S., Seshadri R., Udwary D., Varghese N., Schulz F., Wu D., Paez-Espino D., Chen I.M., Huntemann M. (2021). A genomic catalog of Earth’s microbiomes. Nat. Biotechnol..

[47] Louca S., Parfrey L.W., Doebeli M. (2016). Decoupling function and taxonomy in the global ocean microbiome. Science.

[48] Hyatt D., Chen G.L., Locascio P.F., Land M.L., Larimer F.W., Hauser L.J. (2010). Prodigal: prokaryotic gene recognition and translation initiation site identification. BMC Bioinf..

[49] Parks D.H., Imelfort M., Skennerton C.T., Hugenholtz P., Tyson G.W. (2015). Assessing the quality of microbial genomes recovered from isolates, single cells, and metagenomes. Genome Res..

[50] Kanehisa M., Sato Y., Kawashima M., Furumichi M., Tanabe M. (2016). KEGG as a reference resource for gene and protein annotation. Nucleic Acids Res..

[51] Aramaki T., Blanc-Mathieu R., Endo H., Ohkubo K., Kanehisa M., Goto S., Ogata H. (2020). KofamKOALA: KEGG Ortholog assignment based on profile HMM and adaptive score threshold. Bioinformatics.

[52] Eddy S.R. (2011). Accelerated profile HMM searches. PLoS Comput. Biol..

[53] Virtanen P., Gommers R., Oliphant T.E., Haberland M., Reddy T., Cournapeau D., Burovski E., Peterson P., Weckesser W., Bright J. (2019). SciPy 1.0–Fundamental Algorithms for Scientific Computing in Python. Nat. Methods.

