# Supplementary material for: National antibiotic consumption is strongly related to the prevalence of antibiotic resistance across bacterial clades
Source: iScience. 2025 Jan 6;28(2):111712. doi: 10.1016/j.isci.2024.111712 (PMC11787492; doi:10.1016/j.isci.2024.111712)
Supplement: Document S1. Figures S1–S7 and Table S1 [file mmc1.pdf]

iScience, Volume *28*

Supplemental information

National antibiotic consumption is strongly related to the prevalence of antibiotic resistance across bacterial clades

Stilianos Louca

Supplemental Information

Table S1: **Antibiotic resistance genes, related to STAR Methods.** Overview of antibiotic resistance genes detected and examined (types and KEGG KO numbers). For gene names and descriptions see Data S2.

**Type**	**KOs**
aminoglycoside resistance	K00662, K00663, K00897, K00984, K03395, K04343, K05593, K10673, K12570, K17840, K17880, K17881, K17882, K17910, K18815, K18816, K18817, K18844, K18845, K18846, K19272, K19273, K19274, K19275, K19276, K19278, K19279, K19300, K19301, K19883
beta-lactamases	K01467, K17836, K17837, K17838, K18698, K18699, K18767, K18768, K18780, K18781, K18782, K18790, K18791, K18792, K18793, K18794, K18795, K18796, K18797, K18970, K18971, K18973, K18976, K19095, K19096, K19097, K19100, K19101, K19209, K19210, K19211, K19212, K19213, K19214, K19215, K19216, K19217, K19218, K19316, K19317, K19318, K19319, K19320, K19321, K19322, K20319, K20320, K21266, K21276, K21277, K22232, K22331, K22333, K22334, K22335, K22351, K22352, K24153, K24161, K24162
fosfomycin resistance	K11210, K21252, K21253, K21264, K21265
Macrolide-Lincosamide-Streptogramin (MLS) resistance	K00561, K06880, K06979, K08160, K08217, K15632, K18230, K18231, K18232, K18234, K18235, K18236, K18833, K19349, K19350, K19545, K21251
phenicol resistance	K00638, K07552, K18552, K18553, K18554, K19271
quinolone resistance	K08167, K18555
rifamycin	K19062, K21267, K21288, K22579
sulfonamide resistance	K18824, K18974
tetracycline resistance	K08151, K08168, K18214, K18215, K18216, K18217, K18218, K18220, K18221, K18233
trimethoprim resistance	K18589, K18590, K18591, K19645
vancomycin resistance	K18345, K18344, K07260, K18346, K18351, K18352, K18353, K18347, K15739, K08641, K18350, K18349, K18348, K18856, K18866

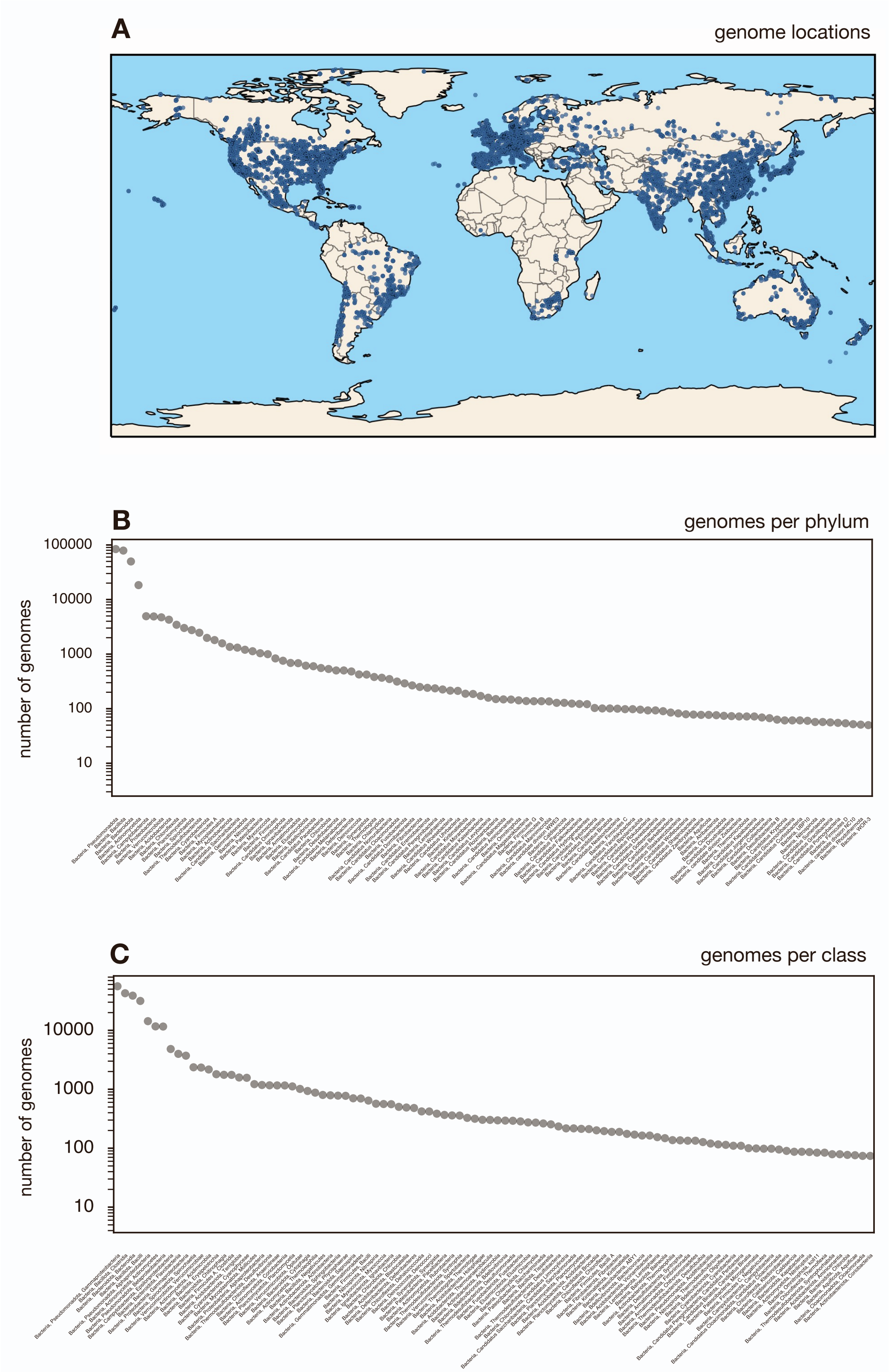

Figure S1: **Overview of genomes, related to STAR Methods.** (A) Geographic locations of genomes for which coordinates were available (109,223 out of 300,209 genomes). (B & C) Number of genomes analyzed per phylum or class, respectively, only showing the top 100 most represented phyla or classes.

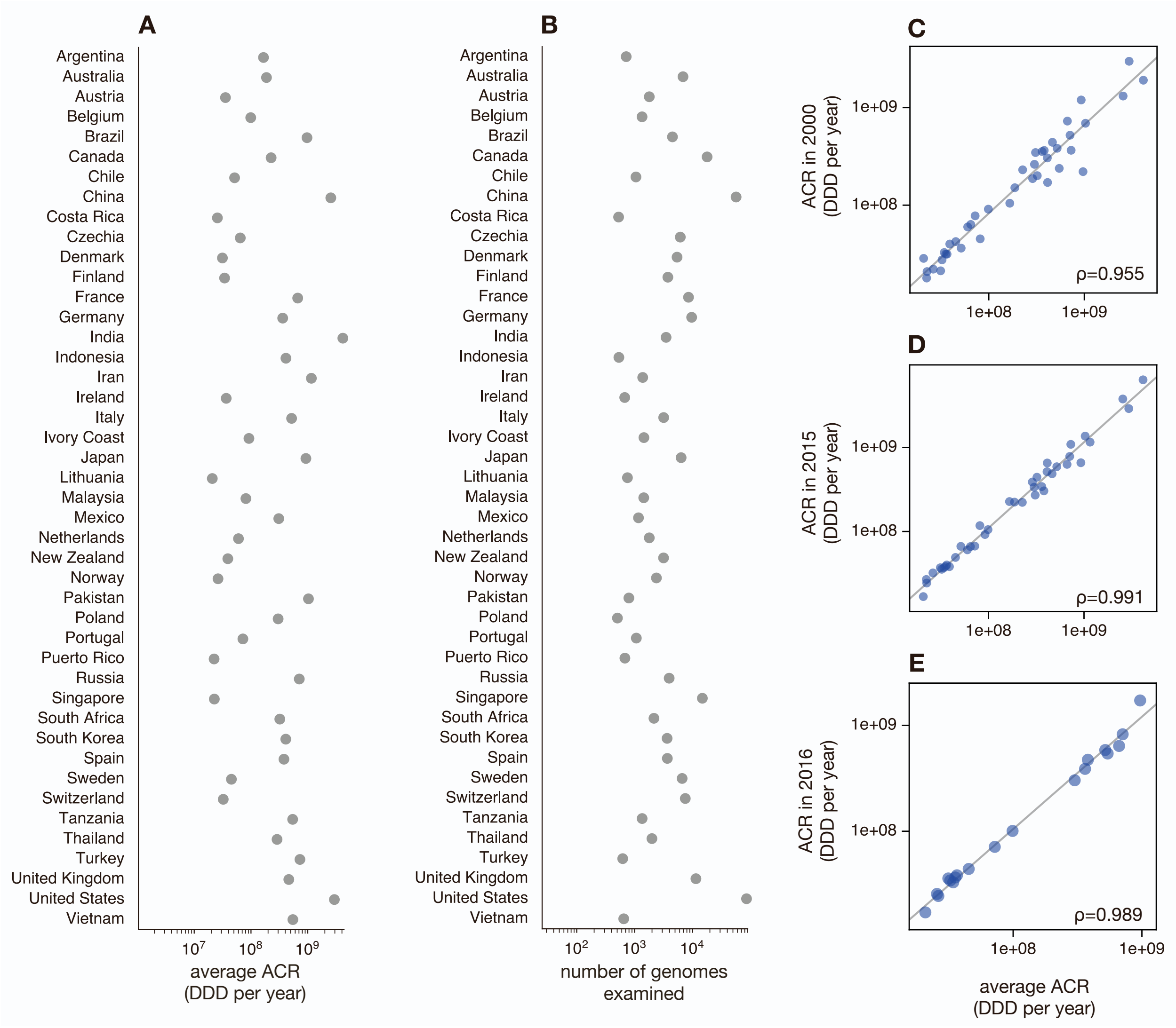

Figure S2: **Antibiotic consumption rates, related to STAR Methods.** (A) National antibiotic consumption rate (ACR) for each considered country, estimated average during years 2000–2016. (B) Numbers of genomes analyzed per country. (C) Comparison of the ACR in year 2000 (vertical axis) to the estimated average ACR during years 2000–2016 (horizontal axis). (D & E) Similar to C, but for years 2015 and 2016, respectively. For detailed country metadata see Data S3.

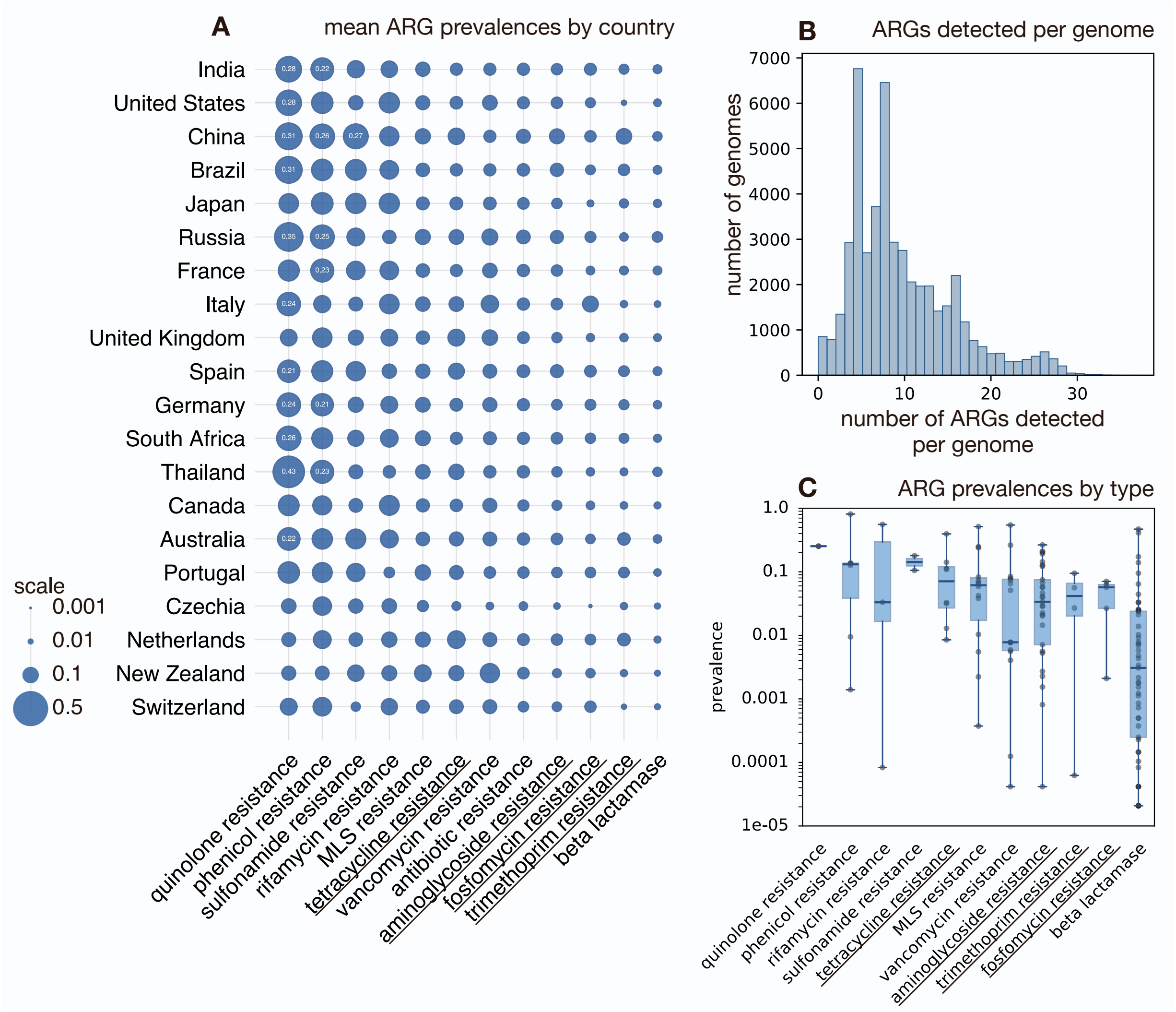

Figure S3: **ARG prevalences in human pathogens, related to Figure 1.** (A) Mean estimated prevalences of antibiotic resistance genes (ARG) in analyzed bacterial strains identified as human pathogens, for each country (rows) and each gene type (columns). Circle sizes are proportional to mean ARG prevalences, i.e., the fraction of cells exhibiting any given ARG averaged over all ARGs of a specific type. Values above 0.2 are written inside the circles. ARG types conferring resistance to antibiotics classified as “reserve” by the WHO AWaRe system are underlined. (B) Histogram of the number of ARGs detected in individual genomes, not adjusted for genome completeness (each genome is one data point). (C) Estimated prevalences of individual ARGs by type (each point is one gene, each box represents one gene type). Whiskers show the full value range, boxes show interquartile ranges, horizontal lines show medians. Gene types are sorted by mean prevalence.

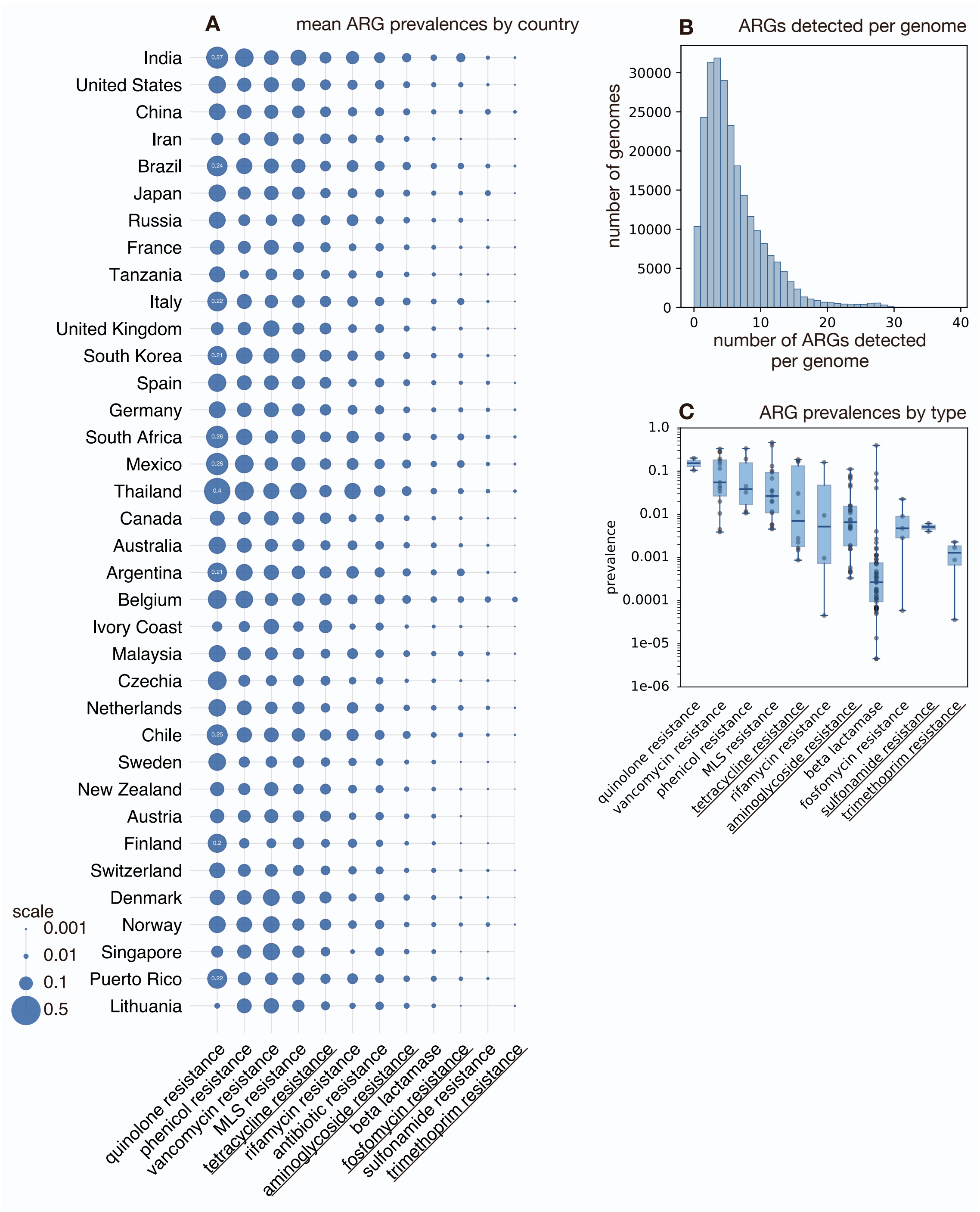

Figure S4: **ARG prevalences in non-pathogens, related to Figure 1.** (A) Mean estimated prevalences of antibiotic resistance genes (ARG) in analyzed bacterial strains not identified as human pathogens, for each country (rows) and each gene type (columns). Circle sizes are proportional to mean ARG prevalences, i.e., the fraction of cells exhibiting any given ARG averaged over all ARGs of a specific type. Values above 0.2 are written inside the circles. ARG types conferring resistance to antibiotics classified as “reserve” by the WHO AWaRe system are underlined. (B) Histogram of the number of ARGs detected in individual genomes, not adjusted for genome completeness (each genome is one data point). (C) Estimated prevalences of individual ARGs by type (each point is one gene, each box represents one gene type). Whiskers show the full value range, boxes show interquartile ranges, horizontal lines show medians. Gene types are sorted by mean prevalence.

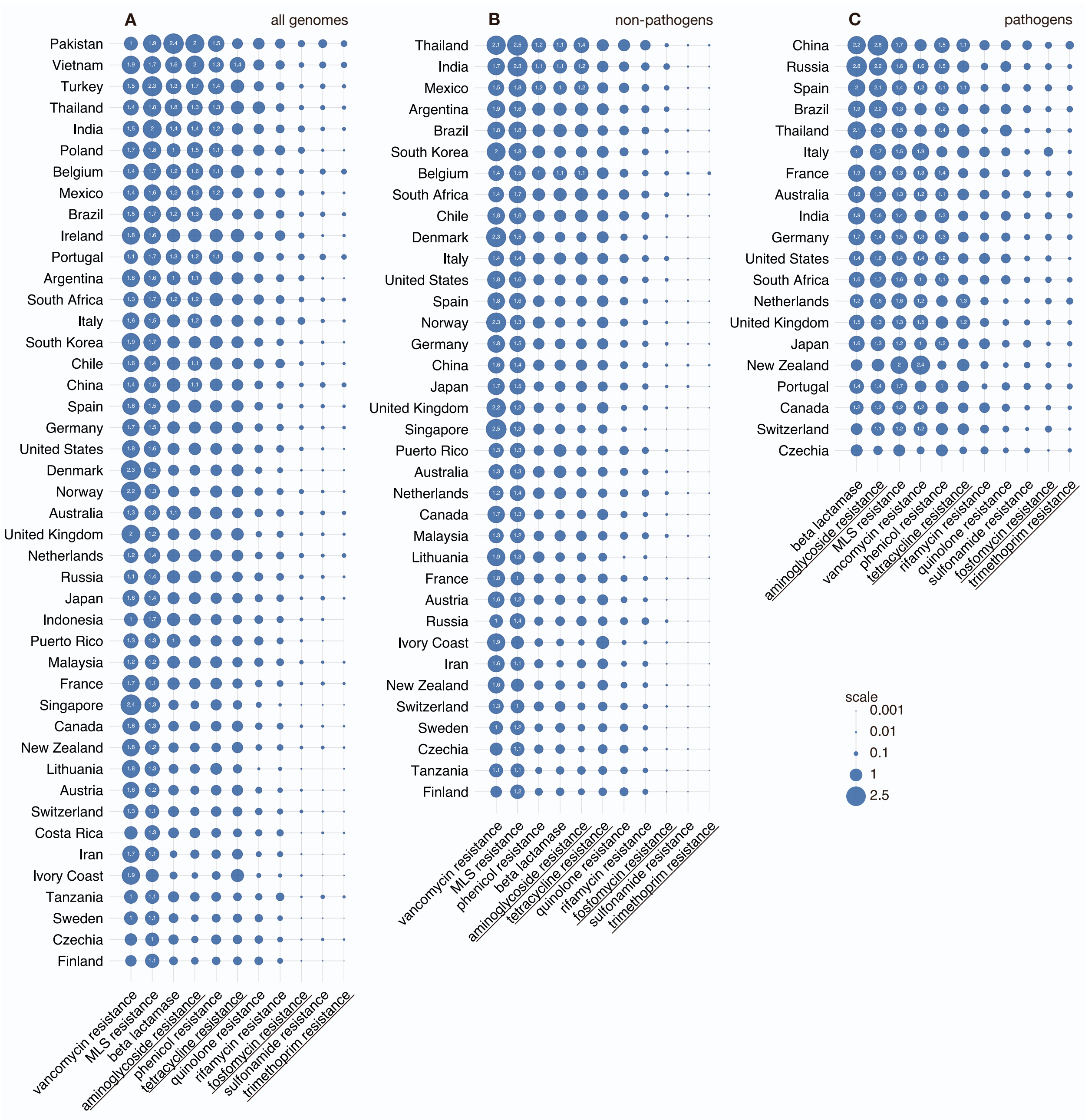

Figure S5: **ARG prevalences (sums), related to Figure 1.** (A) Sum of estimated prevalences of antibiotic resistance genes (ARG) in analyzed bacterial strains, for each country (rows) and each gene type (columns). Circle sizes are proportional to sum of ARG prevalences, i.e., the expected total number of ARGs of a specific type per genome. Values above 1 are written inside the circles. Countries are sorted based on mean circle size. ARG types conferring resistance to antibiotics classified as "reserve" by the WHO AWaRe system are underlined. (B) Similar to A, but restricted to genomes not identified as human pathogens. (C) Similar to A, but restricted to genomes identified as human pathogens. Since only a subset of countries exhibited a sufficient number of non-pathogen genomes for analysis, not all countries from A are included in B; similarly for C.

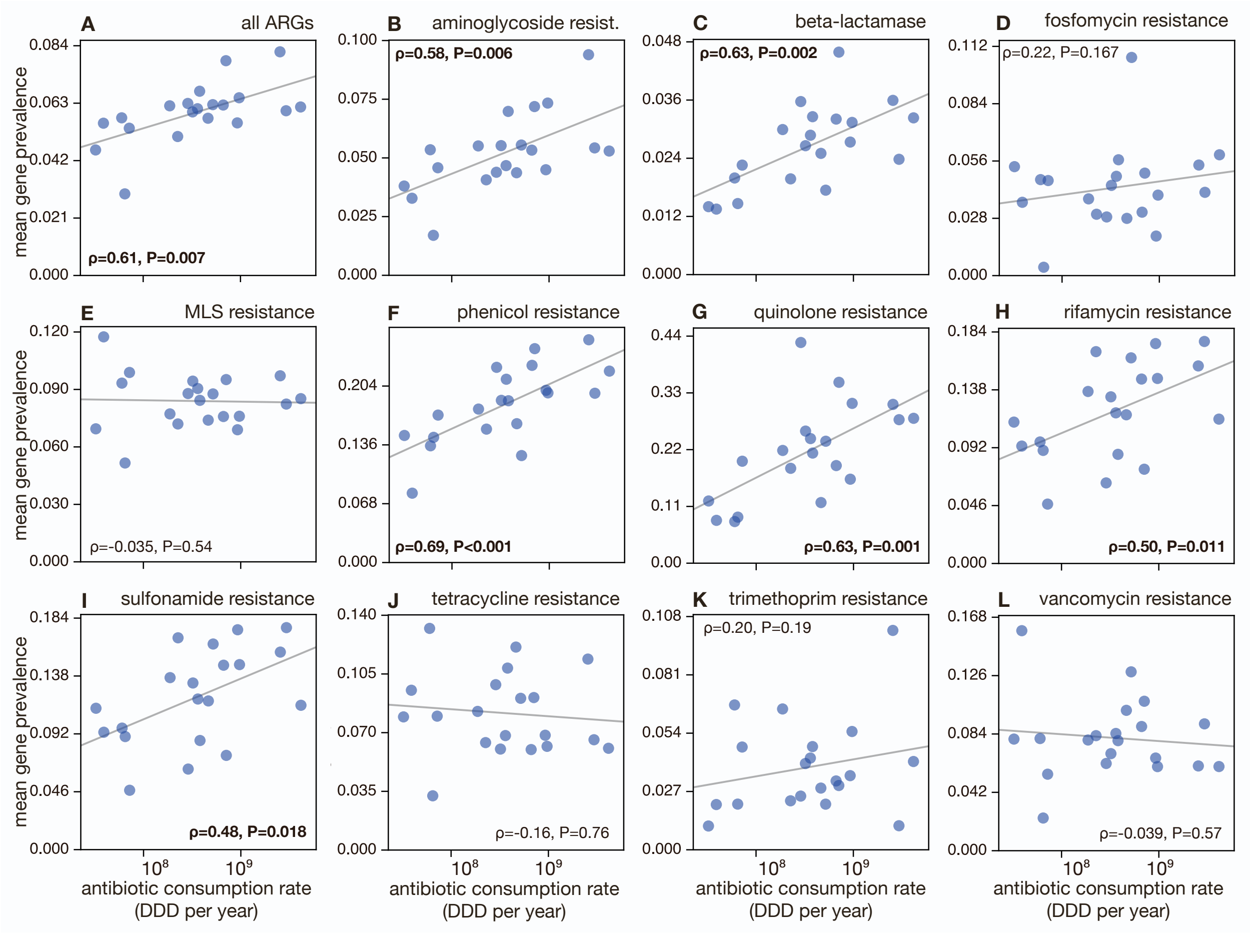

Figure S6: **ARG prevalence in pathogens vs ACR, related to Figure 2.** (A) Mean estimated ARG prevalence in identified human pathogens (fraction of cells exhibiting a given gene, averaged over all genes, vertical axis) compared to national antibiotic consumption rate (ACR, daily dose equivalents per year, horizontal axis) across 20 countries (one point per country). Note that since only a subset of genomes is considered (i.e., only those of human pathogens), some countries with an insufficient number of genomes (<500 genomes) were excluded from the plot. (B–L) Similar to (A), but focusing on specific categories of ARGs. In all figures, the Spearman rank correlation (ρ) and its associated one-sided statistical significance (P) are shown (bold letters highlight statistically significant cases). Log-linear least-squares regression lines are shown for reference.

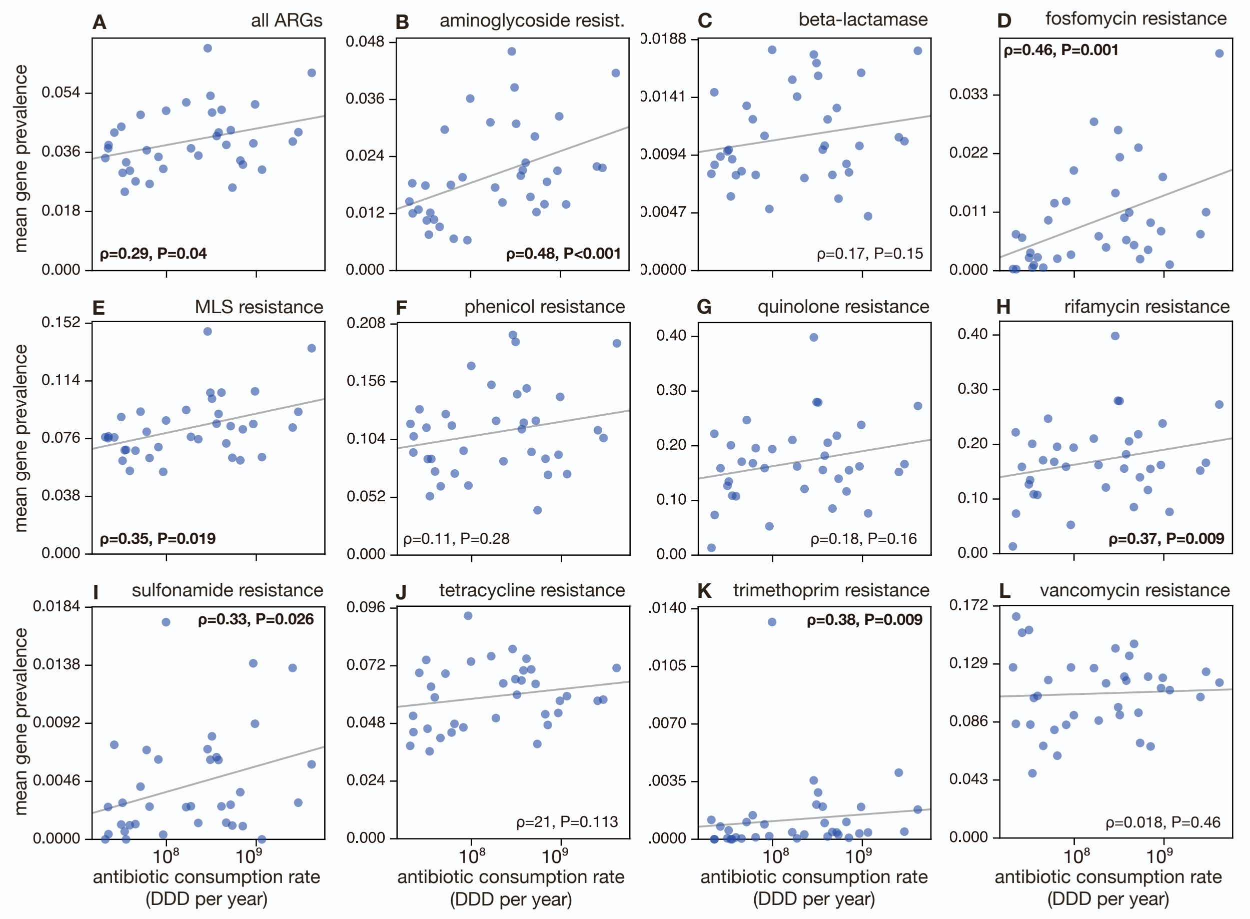

Figure S7: **ARG prevalence in non-pathogens vs ACR, related to Figure 2.** (A) Mean estimated ARG prevalence in strains not identified as human pathogens (fraction of cells exhibiting a given gene, averaged over all genes, vertical axis) compared to national antibiotic consumption rate (ACR, daily dose equivalents per year, horizontal axis) across 36 countries (one point per country). Note that since only a subset of genomes is considered (i.e., only those not from human pathogens), some countries with an insufficient number of genomes (<500 genomes) were excluded from the plot. (B–L) Similar to (A), but focusing on specific categories of ARGs. In all figures, the Spearman rank correlation (ρ) and its associated one-sided statistical significance (P) are shown (bold letters highlight statistically significant cases). Log-linear least-squares regression lines are shown for reference.
